# PestRefineDet: integrating DenseNet with RefineDet for precision pest detection in agriculture

**DOI:** 10.3389/fpls.2026.1921319

**Published:** 2026-08-05

**Authors:** Saleh Albahli, Tahira Nazir, Marriam Nawaz

**Affiliations:** 1 Department of Information Technology, College of Computer, Qassim University, Buraydah, Saudi Arabia; 2 Faculty of Computing, Riphah International University, Islamabad, Pakistan; 3 Department of Software Engineering, University of Engineering and Technology-Taxila, Taxila, Pakistan

**Keywords:** agriculture, computer vision, deep learning, image processing, pest classification

## Abstract

**Introduction:**

Insect pests are a major threat to agricultural productivity, causing significant crop losses and reducing food production. Accurate and timely pest detection is essential for minimizing economic losses and supporting sustainable crop management. However, manual pest identification is labor-intensive, time-consuming, and challenging due to the high visual similarity among many pest species and the limited availability of expert knowledge.

**Methods:**

To address these challenges, we propose PestRefineDet, a deep learning-based framework that integrates DenseNet-41 with RefineDet for automated pest detection and classification. DenseNet-41 is employed as the backbone network to enhance feature extraction through dense connectivity, enabling effective learning of fine-grained pest characteristics. The extracted features are subsequently processed by the one-stage RefineDet framework for simultaneous pest localization and classification.

**Results:**

The proposed framework was evaluated on the challenging IP102 benchmark dataset. Experimental results demonstrate that PestRefineDet achieves a mean Average Precision (mAP@0.5) of 83.61%, providing accurate localization and reliable classification of diverse pest species while maintaining computational efficiency.

**Discussion:**

The results indicate that the proposed framework effectively captures discriminative pest features and improves detection performance on a large-scale, real-world pest dataset. PestRefineDet provides a promising solution for automated agricultural pest monitoring and has the potential to support intelligent and sustainable pest management applications.

## 1 Introduction

Crops play a vital role in sustaining life around the globe, serving as the primary source of food, fodder, and raw materials for various industries. They form the foundation of our diets, providing essential nutrients and energy (Chen et al., 2026; Sparks et al., 2026). Moreover, crops contribute substantially to the worldwide economy by supporting the incomes of masses of people working in the area of agriculture (Ayaz and Mughal, 2024). As a cornerstone of our survival and well-being, the health and productivity of crops are of utmost importance (Amiri and Bandani, 2026). However, numerous factors threaten the health of crops, with pests standing out as a major adversary (Misra et al., 2024). Pests, ranging from insects to diseases, can cause substantial harm by feeding on crops, transmitting diseases, and compromising overall plant health. This poses a significant challenge to farmers by resulting in reduced yields, monetary damages, and possible food deficiency (Kannan et al., 2023; Venkateswara and Padmanabhan, 2025). Further, other factors like adverse weather conditions, soil degradation, and water scarcity also challenge the agriculturist; however, the damage caused by crop pests is more prominent (Grele and Richards, 2026). Therefore, timely detection of pests emerges as a critical strategy in safeguarding crops from these threats. Detecting pests early allows for swift and targeted intervention, preventing the escalation of damage and minimizing the use of harmful pesticides. Early identification enables farmers to devise effective control procedures to ensure the health and productivity of crops while minimizing economic losses (Deguine et al., 2023; Ragu and Teo, 2025). In many parts of the world, especially in economically disadvantaged regions, the challenges in protecting crops are compounded by limited resources and financial constraints (Bellone et al., 2023). Pesticides and advanced pest control methods can be prohibitively expensive for farmers in poorer countries. Additionally, the lack of access to modern agricultural technologies further exacerbates the difficulties in implementing effective pest management strategies. The financial burden associated with purchasing pesticides and adopting advanced methods creates a barrier for farmers in low-income countries. As a result, they may resort to less effective or outdated pest control measures, which often prove to be insufficient in preventing significant crop losses. The disparity in access to resources underscores the need for affordable and sustainable pest management solutions tailored to the specific challenges faced by farmers in economically disadvantaged regions (Daelemans et al., 2023). Timely detection of pests becomes even more crucial in these contexts, as it allows for the early implementation of cost-effective and environmentally friendly control measures. By addressing the economic constraints faced by farmers in poorer countries and promoting accessible technologies, we can work towards ensuring food security and sustainable agriculture on a global scale (Tortosa et al., 2023).

Current pest recognition approaches heavily depend on manual procedures, where farmers and agrarian workers visually inspect crops for signs of pest infestations. However, this traditional approach presents significant limitations. Firstly, manual detection is inherently subjective and open to human error, potentially leading to misidentifications and inaccurate pest assessments. This lack of precision compromises the overall effectiveness of pest control measures (Choe et al., 2023). Moreover, the time-consuming nature of manual inspection poses challenges, particularly in large agricultural areas. Farmers often need to allocate substantial time and labor resources to cover extensive fields, introducing delays in the identification of pest threats. In agriculture, where timely interventions are crucial, such delays can result in increased damage and reduced crop yields. The scalability of manual pest detection becomes increasingly challenging as farms expand in size and complexity. The human workforce may struggle to keep pace with the demands of extensive agricultural operations, leaving certain areas vulnerable to undetected pest infestations (Teixeira et al., 2023). Additionally, the resource-intensive nature of manual detection, requiring skilled labor for accurate identification, may pose barriers in regions with limited access to trained personnel. To overcome these limitations, there is a persistent requirement to transition towards advanced and automated pest recognition technologies that can offer more precise, effective, and scalable solutions for timely pest detection. Methods like machine learning (ML), remote sensing, and sensor networks were recognized to revolutionize pest detection by tackling the problems of manual methods and enhancing the resilience of global agriculture (Thomas et al., 2023).

Initially, conventional ML approaches were employed for the identification of pests in crops. These methods typically involve the development of models based on predefined features and patterns associated with known pest species. While these approaches marked a significant advancement, they came with certain limitations (Wang et al., 2023). Conventional ML techniques often rely heavily on handcrafted features, which may not capture the full complexity of pest-related patterns in crop data. Designing effective features requires domain expertise, and these features might not generalize well to diverse pest populations and changing environmental conditions. Additionally, conventional ML models might struggle with non-linear relationships and complex interactions within large datasets, limiting their ability to provide accurate and robust pest identification (Nawaz et al., 2024b). Deep learning (DL) has appeared as a transformative method in pest identification, addressing some of the limitations of conventional ML. DL models, particularly neural networks, are capable of computing the complex features and representations from raw data by minimizing the need for manual feature engineering. This adaptability enables DL models to handle more complex and diverse datasets, improving their capacity to recognize subtle patterns associated with different pest species.DL models, such as CNNs for image data or RNNs for sequential data, have demonstrated remarkable success in pest detection tasks (Albahli and Nawaz, 2023). They can efficiently process large volumes of data, capture intricate relationships, and adapt to varying conditions, providing a more accurate and versatile approach to pest identification.

DL has undeniably shown remarkable capabilities, particularly in tasks like pest identification, but it is not exclusive of its challenges. One significant hurdle lies in the demand for substantial labeled data for training accurate and reliable DL models (Nawaz et al., 2023a). Acquiring large, well-annotated datasets for every possible pest species and environmental condition can be impractical, especially in cases where data collection is labor-intensive or time-consuming. Another challenge is the computational resources needed for model training effectively. DL models often comprise complicated constructions with huge parameters, demanding powerful hardware for efficient training (Nawaz et al., 2024a). This requirement poses barriers for farmers or researchers in resource-constrained environments, limiting the widespread adoption of DL technologies in pest identification. Interpreting the decision-making process of deep neural networks is also a challenge. The “black-box” nature of these models means that understanding why a particular decision was made can be difficult. This lack of interpretability can hinder the trust and acceptance of DL models in practical applications, especially in critical domains like agriculture. Given these challenges, there is a pressing need for a more effective strategy that strikes a balance between the remarkable capabilities of DL and the practical constraints faced in real-world scenarios (Nawaz et al., 2023b). This involves developing such a model that combines the strengths of DL with more interpretable and resource-efficient techniques. Additionally, investing in research to improve DL’s efficiency in learning from smaller datasets and optimizing model architectures for better interpretability can contribute to overcoming these challenges. A more effective and practical approach is crucial to ensuring that the benefits of DL in pest identification can be harnessed effectively across diverse agricultural landscapes.

The proposed approach is focused on overcoming the present challenges of pest detection and classification by proposing a more effective and efficient strategy. Clearly, a novel DL framework called PestRefineDet is proposed, integrating DenseNet with RefineDet for precision pest detection and classification in agriculture for sustainable farming. Descriptively, the conventional RefineDet approach is modified by using the DenseNet-41 as its base model to compute the related group of sample attributes, which are then classified by the 1-stage detection and classification module of the RefineDet approach. By incorporating DenseNet, which establishes dense connections between layers, the model can leverage comprehensive feature extraction, capturing intricate details in pest images. The synergy between RefineDet’s object detection capabilities and DenseNet’s feature-rich representations enhances the model’s capacity to discern subtle characteristics crucial for pest identification. This customized architecture provides an effective and efficient solution for perceiving and categorizing pests in agricultural contexts, promoting accurate results and facilitating the deployment of advanced pest management strategies. We have evaluated the approach on a complex and larger data sample of pests called IP-102, comprising 102 types of crop pests, and proved the efficacy of our approach. The key findings of our proposed work are:

- We propose PestRefineDet, an effective one-stage pest detection framework that integrates DenseNet-41 as the backbone of RefineDet to enhance discriminative feature representation for fine-grained agricultural pest detection while maintaining computational efficiency.
- We improve pest localization and classification by exploiting dense feature reuse and multi-scale feature propagation, enabling more accurate detection of small and visually similar pest species under complex imaging conditions.
- We conduct a comprehensive experimental evaluation on the challenging IP102 benchmark, including localization, classification, and comparative analysis with state-of-the-art methods, and ablation studies, demonstrating the effectiveness of the proposed framework.
- The proposed model contributes to advancements in sustainable pest management practices. By enhancing precision, the model facilitates targeted interventions, minimizing the need for widespread pesticide use. This, in turn, aligns with sustainable agricultural practices, promoting environmentally friendly and resource-efficient pest management strategies.

The latter paper is organized into distinct sections to provide a structured presentation of the research. Section 2 encompasses a comprehensive literature review, surveying existing works in pest detection and classification to establish the context and highlight gaps addressed by the current study. Moving forward, Section 3 details the methodology, explicating the integration process of DenseNet with RefineDet. Following this, Section 4 outlines the experimental setup, emphasizing the utilization of the IP102 dataset, and is dedicated to presenting the results, including quantitative metrics and visualizations of the model’s performance. Finally, Section 5 serves as the conclusion, summarizing key contributions, implications of the findings, and proposing directions for future research. This organized structure guides readers through the logical progression of the research, ensuring clarity and coherence in presenting the methodology, results, and discussions.

## 2 Related work

This part of the manuscript holds a detailed review of approaches presented for the detection and classification of pests from plants.

Wang et al. (2024) proposed a DL approach called the dilated multi-scale attention U-Net (DMSAU-Net) approach designed to locate crop pests from field plants. The approach employed dilated Inception as the base network in the encoder part of the U-Net to compute the dense visual information of insect pests. Further, an attention unit was introduced in the decoder unit to emphasize the contour of the pests from the investigated images and minimize the upsampling clutter. The model performs well for segmenting insect pests from given images; however, it requires optimization to be employed for real-world cases. In (Toscano-Miranda et al., 2024), a DL model was proposed where three types of transfer learning approaches, called instance, feature, and parameter-based, were employed over the CNN model for crop pests classification. The approach reports the highest results for an instance-oriented transfer learning approach with an accuracy value of 90.79%; however, results need further enhancement. Guo et al. (2024) proposed a lightweight model for the recognition of plant pests from digital images. For this, the work utilized the ResNet-8 approach as an end-to-end framework for calculating dense features and accomplishing the classification task. Further, the model was modified by proposing a temperature-scaled cross-entropy loss method. The work shows better classification results; however, recognition power requires further improvement. Dong et al. (2024) introduced an approach called PestLite in which an object detection approach called YOLO-5 was employed. The work has modified the conventional architecture of the YOLO-5 approach’s attention mechanism to focus on the targeted areas. The approach shows improved pest detection results; however, unable to locate pests of small sizes. Another such model was suggested in (Sun et al., 2024), where an optimized YOLOv5s framework was suggested to overcome the complexities of plant pest recognition. The approach added Channel Attention (CA) units to enhance the computation of dense features. Framework convergence was optimized by employing the K-means++ approach to focus on the targeted regions. The Efficient Intersection over Union (EIOU) loss method was utilized to tackle unstable sample labels. The technique shows effective pest recognition results; however, unable to deal with sample transformation changes. Hassan et al (Hassan and Maji, 2024). proposed a DL approach for classifying crop pests from field images. The work tested various variants of the ResNet approach, like 50, 101, and 152-layered architectures, by adding a self-attention mechanism in them and tuning them in an end-to-end manner. Among all architectures, the modified ResNet-50 approach performs well, however, the model is unable to locate the exact location of pests.

Chodey et al (Chodey and Shariff, 2023). proposed an approach for recognizing pests from investigated images. First, a phase was used to preprocess the samples to enhance the pictorial depictions of the samples. Next, the Fuzzy C-means (FCM) approach was applied to cluster the focused regions. Next, the features from the segmented part of the images were applied using the GLSM approach to capture the textural information. Finally, for classifying the samples, the LSTM module was used. This approach shows improved pest classification results; however, the model may not perform well in classifying the distorted samples. In (Prasath and Akila, 2023), an object recognition framework has been investigated to locate and categorize plant pests. For this reason, the YOLO-V3 approach was utilized through an optimization approach called the Adaptive Energy-based Harris Hawks Optimization technique. For computing the visual information of the suspected images, two DL models named the VGG-16, and ResNet50 were used. The computed information from the feature extractor was then recognized by the 1-phase locator of the YOLO-V3 approach. The approach shows an efficient solution for pest recognition; however, detection results need improvement. Another such framework was discussed in (Tang et al., 2023), where an upgraded version of the YOLO model was introduced, focusing on increasing accuracy in real-time pest recognition. The improved Pest-YOLO merged 2 major changes: an efficient channel attention (ECA) strategy to gather refined visual information and the inclusion of a transformer encoder to hold global features. The base squeeze-excitation strategy was substituted with ECA, advancing the framework’s power to nominate crucial characteristics from pest samples. Further, a transformer encoder was proposed for the CNN unit, increasing its competence to gather detailed related information globally. Further, a cross-stage feature fusion (CSFF) approach was adopted to locate the pests of small size. The results were evaluated on the Pest24 data sample with a mean average precision of 73.4%. The approach performs well for pest detection; however, it needs huge training data. Anwara et al (Anwar and Masood, 2023). proposed the concept of an ensemble network for performing the classification of crop pests. The model merged 3 models, named the 16-layered and 19-layered VGG model with the ResNet50 approach. The features from all 3 models were combined to accomplish the classification task. The approach performs well in recognizing the pests of multiple types, however, suffering from a huge computational burden. Ali et al. (2023) proposed a DL approach called the Faster-PestNet by presenting an improved version of the Faster-RNN model to recognize crop pests from given images. Clearly, the MobileNet was utilized as the feature extractor of the original Faster-RCNN model to extract the dense information, which was then recognized through the localization part of the model. The approach improves pest recognition results, however, unable to tackle the distorted samples. In (Yang et al., 2023), another object recognition approach has been explored for the timely identification of crop pests from digital images. The YOLO-7 approach was explored for this purpose by merging the CSPResNeXt-50 unit with VoVGSCSP to improve the detection performance. The approach performs well for pest recognition; however, ineffective in tackling intra-class variations. Shafik et al. (2023) suggested a framework utilizing both the spatial and temporal information of samples to categorize the crop pests from the investigated images. An improved CNN framework together with long short-term memory (LSTM) was utilized to capture the visual information of the pest samples, which were then classified by employing the majority voting classifier. The model was evaluated on Turkey data samples of pests to recognize the insects from the apple plants. The approach performs well for the designated tasks; however, the model needs evaluation on a larger set of pest samples with more diverse data types and environmental conditions.

Another DL framework was discussed in (Venkatasaichandrakanth and Iyapparaja, 2023), where an Enhanced vision transformer architecture (EViTA) approach was proposed for recognizing crop pests. For this, first the moth flame optimization (MFO) approach was utilized to improve the pictorial depiction of samples. Then, the EViTA approach was used for dense features computation and for performing the cataloging of the samples into various types. The work shows enhanced pest recognition results; however, the approach increases the computing burden and is only evaluated for the pests from the peanut crop. Mallick et al. (2023) proposed a DL approach for classifying the mung bean pests. For this reason, the work has utilized the MobileNet-v2 approach in an end-to-end manner for computing dense features and performing the classification task. The approach shows effective results; however, the model requires testing on a larger set of pest samples with more diverse data types and environmental conditions. Another such framework was suggested in (Rimal et al., 2023), where a DL approach called the MobileNet framework was used for classifying numerous types of crop pests. This approach shows an efficient solution for recognizing crop pests; however, recognition accuracy needs further improvements.

Albattah et al. (2023) investigated an object detection approach to localize and recognize the various types of pests from digital images. For this reason, this work has proposed an improved CornerNet approach in which the conventional feature extractor was replaced by the DenseNet-100 to extract the dense information from the samples. Later, the computed features were recognized through the repressor and classifier unit of the model. This approach effectively locates the pests from the noisy samples; however, unable to tackle the huge light alterations. In (Wei et al., 2022), a hybrid approach was developed to recognize pests of various types, in which 2 modules, named the multi-scale feature extraction module (MFE) and the feature extraction module (DFE), were employed to compute a dense set of sample information. In the next step, the information from both modules was merged to develop the final set of features. Finally, the extracted features were passed to the classification module for performing the categorization of pests into related classes. This approach shows effective classification results; however, unable to locate the location of insects on plants. Another work was defined in (Teng et al., 2022), which introduced a novel technique for crop pest detection named the MSR-RCNN, which integrated a Multi-Scale Super-Resolution Feature Enhancement unit to enhance the network’s ability to detect pests in agricultural settings. This unit improved feature descriptions at numerous scales, contributing to the effective recognition of crop pests. The paper focuses on multi-class detection, addressing the challenges posed by diverse pest species. Through experiments and evaluations, the MSR-RCNN model demonstrated notable performance in detecting crop pests across various classes; however, classification results need improvement. Dong et al. (2022) introduced an automated method for crop pest detection with a focus on automatic and multi-scale feature merging. The approach employed advanced techniques to automatically detect and fuse features at multiple scales, enhancing the model’s ability to identify crop pests effectively. By incorporating multiscale feature fusion, the model aimed to capture intricate details related to pest infestations across different scales in agricultural imagery. The work is found useful in locating pests under diverse environmental conditions, however, the recognition ability needs further enhancements. Further, Agrawal et al. (2026) suggested a DL approach for recognizing pests from the input images by utilizing a structured DensNet approach and attained an accuracy value of 82.69%; however, it lacks the ability to locate the exact location of the pests on the samples. Next, in (Wang et al., 2025), an improved EfficientNetb0 approach was presented for pest classification, and attained an accuracy score of 74.06%, which needs further enhancement.

The critical analysis of the approaches has been presented in **Table 1**, from where it can be seen that there is a need for a more effective approach. Even though a huge amount of work has been proposed by the researchers, pest detection and classification techniques in agriculture encounter several generic challenges that span across various methodologies. One prominent issue lies in the inherent diversity of pest species, which may exhibit variations in appearance, size, and behavior. This diversity complicates the development of a one-size-fits-all model, requiring adaptable approaches that can generalize effectively. Additionally, environmental factors, such as varying lighting conditions and complex backgrounds, pose challenges to the robustness and reliability of detection models. Another common concern is the need for large and well-annotated datasets for training, which can be resource-intensive and may not always capture the full spectrum of pest scenarios. Furthermore, the dynamic nature of agricultural landscapes and the real-time requirements for decision-making demand models that can operate efficiently in dynamic and time-sensitive settings. Addressing these generic challenges is crucial for the development of effective and practical pest detection and classification techniques in agriculture.

**Table 1:** Comparative analysis of existing studies that attempted to perform the pest classification task.

Reference	Year	Technique	Dataset	Results	Limitation
(Agrawal et al., 2026)	2026	structured DensNet	IP102	Accuracy= 82.69%	Unable to locate the exact location of the pests on the samples
(Wang et al., 2025)	2025	improved EfficientNetb0	IP102	Accuracy=74.06%	Classification results need further enhancement
(Wang et al., 2024)	2024	DMSAU-Net	14 rice insect pest categories from IP102	Accuracy= 91.20%	• The model needs optimization to be adopted for real-world cases • Only evaluated for the rice crop
(Toscano-Miranda et al., 2024)	2024	Instance-based transfer learning	Custom dataset	Accuracy= 90.79%	Classification results need further enhancement
(Guo et al., 2024)	2024	ResNet-8	D0 dataset	Accuracy= 84.29%	Recognition performance requires improvement
(Dong et al., 2024)	2024	Modified YOLO-5	IP-102	mAP= 57.10%	The model is unable to locate pests of small sizes
(Sun et al., 2024)	2024	Modified YOLO-5s	Orchard pest dataset	mAP = 92.20%	The approach is not effective in tackling the sample transformation changes.
(Hassan and Maji, 2024)	2024	Modified ResNet-50	Crop pests dataset	Accuracy= 96.68%	The model is unable to locate the exact location of pests
(Chodey and Shariff, 2023)	2023	FCM, GLSM, and the LSTM approach	Custom dataset	Accurcy= 96.04%	The model may not perform well in classifying the distorted samples
(Prasath and Akila, 2023)	2023	YOLO-V3	Subset of IP102	Accuracy= 96%	The model’s performance requires improvements
(Tang et al., 2023)	2023	Improved Pest-YOLO	Pest24	mAP =73.4%	The model needs a huge training sample
(Anwar and Masood, 2023)	2023	VGG-16, VGG-19 along with the ResNet-50	IP102	Accurcy= 82.50%	• The approach is computationally inefficient • The model is unable to identify the exact location of pests
(Ali et al., 2023)	2023	Faster-PestNet	IP102	Accurcy= 82.43%	The model is unable to tackle the distorted samples
(Yang et al., 2023)	2023	Improved YOLO-V7	IP102	mAP =76.3%	The model is ineffective in tackling intra-class variations
(Shafik et al., 2023)	2023	CNN/RNN	Turkey pests dataset	Accuracy= 99.20%	• The approach is only evaluated for the apple pests • The model is unable to identify the exact location of pests
(Venkatasaichandrakanth and Iyapparaja, 2023)	2023	EViTA	Custom dataset	Accuracy= 92%	• The approach is only evaluated for the peanut pests • The approach is computationally expensive
(Mallick et al., 2023)	2023	MobileNet-v2	Custom dataset	Accuracy= 93.65%	• The approach is only evaluated for the mung bean pests • The model is unable to identify the exact location of pests
(Rimal et al., 2023)	2023	MobileNet	IP102`	Accuracy= 82.04%	• The model needs performance improvements • The model is unable to identify the exact location of pests
(Albattah et al., 2023)	2023	Custom CornerNet	IP102	Accuracy= 68.74%	• The model needs performance improvements • The model is ineffective in dealing with light variations
(Wei et al., 2022)	2022	CNN	Custom dataset	Accuracy= 98.2%.	• The model is unable to identify the exact location of pests • The approach lacks to perform well for distorted images
(Teng et al., 2022)	2022	MSR-RCNN	LLPD-26	Accuracy= 67.40%	• The classification results required improvements
(Dong et al., 2022)	2022	MCPD-net	MPD2021	mAP = 67.3%	• The recognition ability needs enhancements

## 3 Proposed model: PestRefineDet

**Figure 1:**
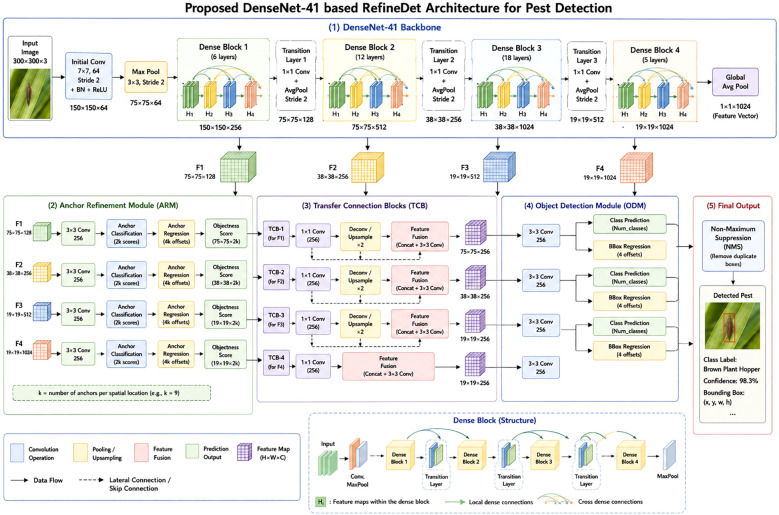
PestRefineDet flow diagram.

Agricultural pest detection is considerably more challenging than generic object detection because pests exhibit small object sizes, complex backgrounds, high inter-class similarity, and significant intra-class variability. Conventional RefineDet employs traditional backbone networks that may not sufficiently preserve low-level discriminative features required for distinguishing visually similar pest species. To address this limitation, we present PestRefineDet, an effective DL model tailored for pest detection and identification. Specifically, DenseNet-41 is incorporated as the feature extraction backbone. Through dense connectivity, DenseNet facilitates feature reuse and improved gradient propagation, enabling richer multi-scale representations that are particularly beneficial for detecting fine-grained pest characteristics. Rather than serving as a simple backbone replacement, this integration is specifically designed to enhance feature representation for agricultural pest detection while retaining the efficient one-stage refinement mechanism of RefineDet. Furthermore, DenseNet-41 was specifically selected to achieve an effective balance between discriminative feature learning and computational efficiency. Unlike deeper backbone networks that increase parameter redundancy and computational burden, DenseNet-41 maintains efficient feature propagation through dense connections while reducing the overall model complexity. This lightweight architecture is particularly advantageous for agricultural applications, where real-time monitoring and deployment on resource-constrained devices are desirable. Therefore, the proposed integration provides not only improved feature representation for fine-grained pest recognition but also a practical and scalable solution for sustainable pest management. PestRefineDet produces numerous bounding boxes and associated confidence values indicating the presence of different pests within those detected regions. The schematic depiction of our model is illustrated in **Figure 1**. Non-maximum suppression is employed to generate the final output. PestRefineDet comprises two interconnected modules: Object Detection (O_D) and Anchor Refinement (A_R). O_D aims to accurately detect and classify various objects based on enhanced anchors, while A_R focuses on eliminating wrong anchors to reduce the exploration space and refine the positions and dimensions of anchors to deliver a beginning for the regressor. The output of Transfer Connection Blocks (TCBs) is merged through forecast layers using a 3 × 3 to form O_D, computes confidence scores for region labels, and forms offsets relative to enhanced anchor box assessment. The primary components of the proposed RefineDet include TCB, Two-Step Cascaded Regression (TSCR), and Negative Anchor Filtering (N_A Filtering).

### 3.1 Transfer connection blocks

The TCBs serve to establish a connection between an O_D and an A_R by transforming structures from several stages within the A_R into the required format for the O_D. This enables the O_D to access features from the A_R unit. Importantly, we exclusively use the TCBs from the A_R to the feature maps associated with anchors. To enhance recognition outcomes, we augment the transferred features with high-level features from the TCBs to integrate large-scale contextual information. We apply a deconvolution approach to generate high-level feature maps, which are then combined element-wise to preserve dimensional consistency. After merging, a convolution layer is introduced to retain the unique characteristics of the features for effective recognition.

### 3.2 TSCR

Existing one-stage methods estimate the positions and sizes of pests through single-stage regression applied to multiple feature layers at different scales. However, such approaches often encounter difficulties in complex situations, particularly when detecting small objects. To overcome these limitations, we introduce a 2-step cascaded regression framework designed to refine both the location and size of detected areas. In order to enhance the initialization of regression within the O_D module, the AR phase is first employed to adjust anchor positions and dimensions. More precisely, each uniformly dispersed cell on the feature map is associated with n anchor boxes. The starting location of every anchor box related to its associated cell remains fixed. We predict 4 adjustments for the refined bounding boxes relative to the original anchors at every feature map cell, along with two confidence scores indicating the presence of foreground objects. Thus, for each cell on the feature map, we generate *n* refined anchor boxes (RABs) and feed them into the feature maps in the O_D module, as depicted in **Figure 1**. The feature maps generated by the O_D and A_R modules share identical dimensions. For the detection process, c class scores and four localization offsets are computed for each region of interest relative to its corresponding refined anchor box, producing a total of c + 4 outputs per anchor. The RefineDet framework follows a two-step pipeline: the A_R module first refines the anchor boxes, and the O_D module subsequently performs detection based on these refined anchors. This hierarchical strategy enhances accuracy, particularly in identifying small objects.

### 3.3 NAF

This unit is applied to eliminate undesirable anchors and mitigate class disparity. During the training phase, for each improved anchor box, the training of the O_D module is skipped if the negative confidence surpasses a predefined threshold (θ = 0.99). As a result, only the hard negative samples and refined positive anchors are retained for training the O_D module, thereby enhancing learning efficiency and detection robustness. Likewise, if a RAB is assigned a negative confidence higher than θ, it is rejected during the identification process within the OD module.

### 3.4 DenseNet-41

To extract deep and optimized features, we utilized the DenseNet-41 model. This technique consists of four Dense Blocks (D_Bs) with the same number of layers as ResNet. Compared to ResNet, DenseNet-41 has fewer parameters, making it more computationally efficient for disease detection feature computation. The D_B is a crucial part of DenseNet, where l × l × m0 represents the key point maps (KM) of the L-1 layer. N indicates the measurement of KM, with m0 characterizing all channels. P(.) denotes the non-linear transformation consisting of various modules such as batch normalization (BaN), ReLU activation, a 1 × 1 Convolutional layer (C_L) for channel reduction, and a 3 × 3 C_L for feature reorganization. Dense connections are depicted by long-dashed arrows, linking layer L-1 to the L layer and integrating them through the result of P(.). Finally, l × l × (m0 + 2m) represents the output of the L + 1 layer.

The various dense links improve Key Metrics (KMs); therefore, T_L is activated to reduce the feature size from the previous Dense Block, as briefly discussed in (Ali et al., 2023; Anwar and Masood, 2023). The estimated features are down-sampled with a stride rate of four, which are then used for estimating different heads, as explained in the following subsections. Feature Maps (FMs) expand due to the extensive dense connections; hence, T_L is employed to minimize the size of the feature map from the preceding D_B.

The detailed configuration of the adopted DenseNet-41 backbone is presented in **Table 2**. The network consists of four dense blocks connected through transition layers. Each dense layer follows the bottleneck design, comprising batch normalization, ReLU activation, a 1×1 convolution for feature compression, and a 3×3 convolution for feature extraction. Dense connections enable effective feature reuse and improved gradient propagation, while transition layers reduce the spatial dimensions and computational complexity of feature maps. The proposed DenseNet-41 contains dense blocks with [3, 6, 6, and 3] bottleneck units, resulting in a total network depth of 41 layers. The extracted multi-scale feature maps are subsequently forwarded to the ARM and ODM modules of RefineDet for object localization and pest classification.

**Table 2:** Configuration of the DenseNet-41 backbone used in PestRefineDet.

Block	Layer type	Configuration	Stride
1	Convolution	7×7 Conv, 64 filters	2
2	Pooling	3×3 Max Pool	2
3	Dense Block 1	(BN-ReLU-1×1 Conv-BN-ReLU-3×3 Conv) ×3	1
4	Transition Layer 1	1×1 Conv + 2×2 Avg Pool	2
5	Dense Block 2	(BN-ReLU-1×1 Conv-BN-ReLU-3×3 Conv) ×6	1
6	Transition Layer 2	1×1 Conv + 2×2 Avg Pool	2
7	Dense Block 3	(BN-ReLU-1×1 Conv-BN-ReLU-3×3 Conv) ×6	1
8	Transition Layer 3	1×1 Conv + 2×2 Avg Pool	2
9	Dense Block 4	(BN-ReLU-1×1 Conv-BN-ReLU-3×3 Conv) ×3	1

### 3.5 Anchor design and matching

In our approach, four feature layers with stride sizes of 8, 16, 32, and 64 pixels are integrated into DenseNet-41 to support multi-scale prediction anchors for objects of varying sizes. Each feature layer is assigned three aspect ratios and a defined set of anchor scales (0.5, 1.0, and 2.0). A uniform anchor scaling strategy is applied across all layers to maintain consistent tiling density across the input samples. The entire model is tuned in an end-to-end manner, where anchors are matched to ground-truth boxes based on their Jaccard overlap values.

### 3.6 Hard negative mining

Despite the exclusion of some straightforward negative anchors from the AR process within the OD, a significant portion of anchors remains negative following the matching phase. To alleviate this imbalance, we implement hard negative mining, a technique utilized in SSD (Prasath and Akila, 2023). The objective is to reduce the maximum disparity between foreground and background categories. Precisely, we select a subset of negative anchors based on the peak calculated loss numbers, aiming to decrease the negative-to-positive ratio to below 3:1. This approach contrasts with randomly selecting negative anchors during training.

### 3.7 Loss method

The loss method of our approach contains two parts: the A_R module loss and the O_D module loss. In the A_R module, each anchor is assigned a binary class tag, and its location and size are simultaneously regressed to generate refined anchors. These refined anchors, with negative values under a specified threshold, are then passed to the O_D module to improve both object classification and accurate localization. The loss function is defined in **Equation 1**:

(1) $$L\left(\left\{p_{i}\right\},\left\{x_{i}\right\},\left\{c_{i}\right\},\left\{t_{i}\right\}\right)=\frac{1}{N_{ar}}\left(\sum_{i}L_{b\left(p_{i},\left[l_{i}\geq 1\right]\right)}\right)+\sum_{i}\left[l_{i}\geq 1\right]L_{r}\left(x_{i},g_{i}^{*}\right))+\frac{1}{N_{od}}\left(\sum_{i}L_{m\left(c_{i},l_{i}\right)}\right)+\sum_{i}\left[l_{i}\geq 1\right]L_{r}\left(t_{i},g_{i}^{*}\right))$$

where:

Here, *i* represents the index of an anchor; *l_i_* denotes the ground-truth category of anchor *i*, and $g_{i}^{*}$ represents its ground-truth position and size. In the A_R module, *p_i_* and *x_i_* correspond to the computed confidence that anchor *i* contains a target and its improved directions, respectively. In the O_D module, *c_i_* and *t_i_* represent the predicted object category and bounding box parameters.

*N_ar_​* and *N_od_​* represent the total positive anchors in the AR and OD, respectively. *L_b_*​ represents the binary categorization loss, *L_m_*​ represents the multi-class classification loss. The function · denotes the indicator function, where *l_i_​*≥1 is true if *i* greater or equals 1, otherwise it equals 0.

### 3.8 Inference phase

This part refines the locations and measurements, discarding anchors that are frequently tiled and have negative confidence scores exceeding the threshold during inference. The O_D module then processes these refined anchors, producing the top 400 most confident detections per image. To generate the final recognition results, non-maximum suppression is applied with a Jaccard overlap threshold of 0.45 per group, retaining the 200 highest-confidence detections for each sample.

## 4 Proposed method results

### 4.1 Dataset

**Figure 2:**
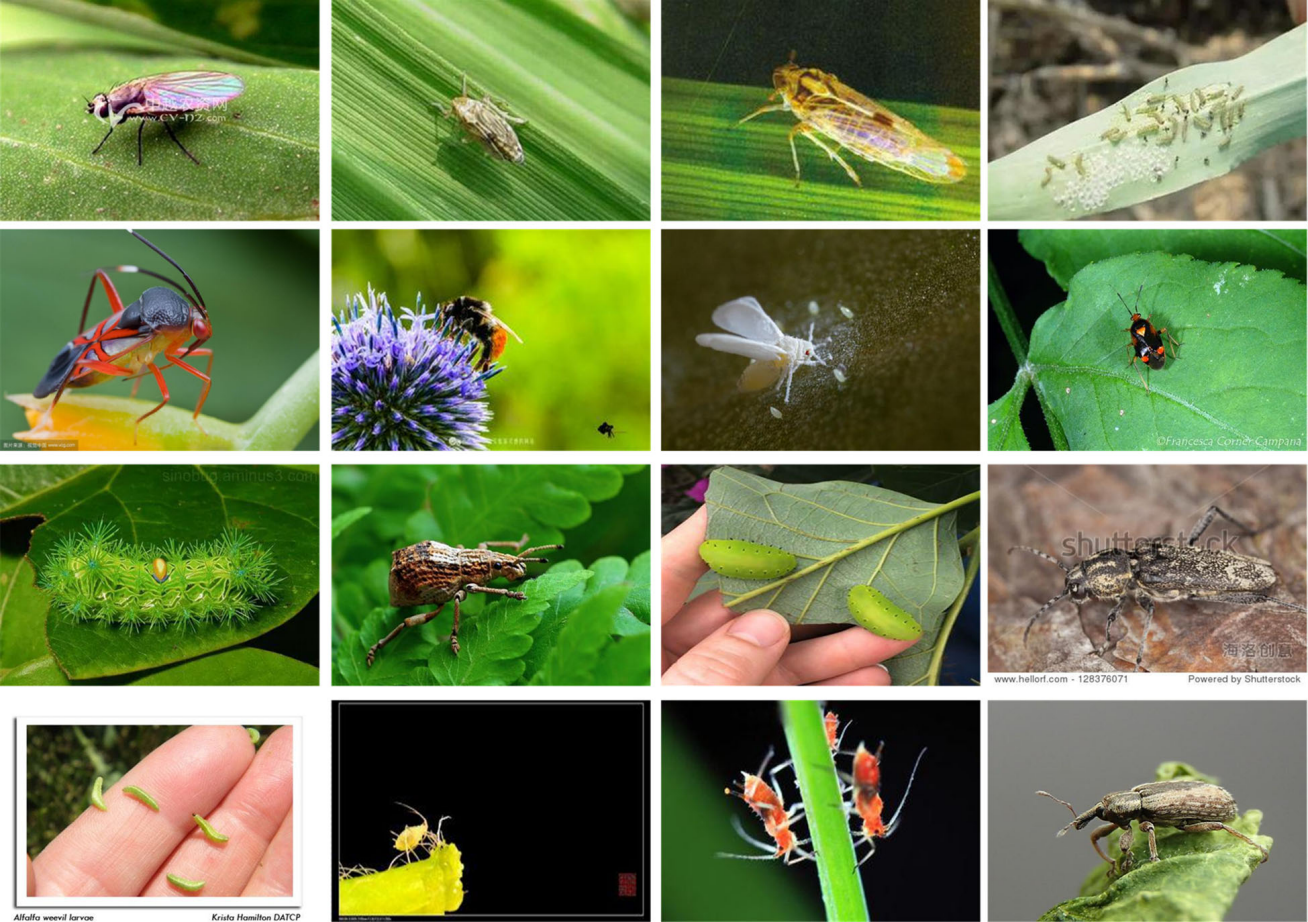
Pest images from the IP102 dataset.

For network training and evaluation, the IP102 dataset is opted for, which is a comprehensive benchmark specifically created for pest recognition from images. This dataset includes 102 groups of crop pests usually found in agricultural fields. IP102 presents considerable challenges for image classification due to its diverse variations in perspective, scale, orientation, and lighting conditions. The dataset consists of 75, 222 RGB images with a resolution of 224 × 224 pixels. Following the official IP102 benchmark protocol, the dataset is divided into training, validation, and testing subsets containing 45, 095, 7, 508, and 22, 619 images, respectively. The predefined split was maintained throughout all experiments, including model training, evaluation, and comparison with baseline methods, to ensure fair and reproducible performance assessment. Additionally, all images are meticulously annotated by human experts at the object level, with one or more pests manually identified in each image. This dataset (samples in **Figure 2**) serves as a challenging standard for pests because of various distinguishing features:

- Diverse types of pests: The IP102 dataset encompasses a wide array of pest categories, highlighting the diversity of pests encountered in agricultural settings.
- Occlusion and clutter: Many images within this dataset exhibit occlusions and clutter, like numerous objects in the scene or pests partly hidden by other elements.
- Imbalanced class distribution: The distribution of images across categories within the IP102 dataset is imbalanced, where some classes contain only a few samples while others contain thousands.

These unique characteristics make the IP102 dataset a valued resource for assessing the performance of pest recognition and detection models.

### 4.2 Implementation details

The proposed PestRefineDet framework was implemented using Keras with TensorFlow as the backend. The experiments were conducted on a workstation equipped with an NVIDIA RTX 3090 GPU (24 GB memory), 32 GB RAM, and Windows 10 operating system. The software environment consisted of Python 3.8, TensorFlow 2.10, CUDA 11.2, and cuDNN 8.1. The DenseNet-41 backbone was initialized using ImageNet pretrained weights, and the complete network was fine-tuned on the IP102 dataset. The model was optimized using the Stochastic Gradient Descent (SGD) optimizer with a learning rate of 0.0015, momentum of 0.9, and weight decay of 0.0005. Training was performed for 30 epochs with a batch size of 32. The input images were resized to 320 × 320 pixels and normalized before training. No additional data augmentation was applied to evaluate the inherent feature learning capability of the proposed framework. Following the official IP102 benchmark protocol, the predefined training, validation, and testing subsets were used without additional random splitting, consisting of 45, 095, 7, 508, and 22, 619 images, respectively. The same split was used for the proposed model, baseline comparisons, and ablation experiments to ensure fair evaluation. The proposed framework follows the original RefineDet architecture, including the ARM and ODM. The default anchor generation and matching strategy of RefineDet was adopted, where anchors with IoU ≥ 0.5 were assigned as positive samples and anchors with IoU< 0.4 were considered negative samples. The training objective follows the original RefineDet multi-task loss formulation, combining localization regression and classification losses with the default loss weights. A fixed random seed of 42 was used for reproducible model initialization and data processing. All experiments were performed under identical settings, and the reported results represent the final model performance evaluated on the official IP102 test set. For object localization, the original RefineDet anchor generation strategy was maintained. Anchors were generated at multiple feature scales using three aspect ratios (1:1, 1:2, and 2:1). The anchor scales were distributed across feature layers from 0.10 to 0.90 relative to the input image size, enabling the detection of pests with different spatial dimensions. The proposed PestRefineDet contains approximately 11.4 million trainable parameters and requires around 3.1 GFLOPs for an input resolution of 320 × 320 pixels. The complete training process required approximately 14 hours for 30 epochs on the NVIDIA RTX 3090 GPU.

### 4.3 Evaluation of pest recognition

**Figure 3:**
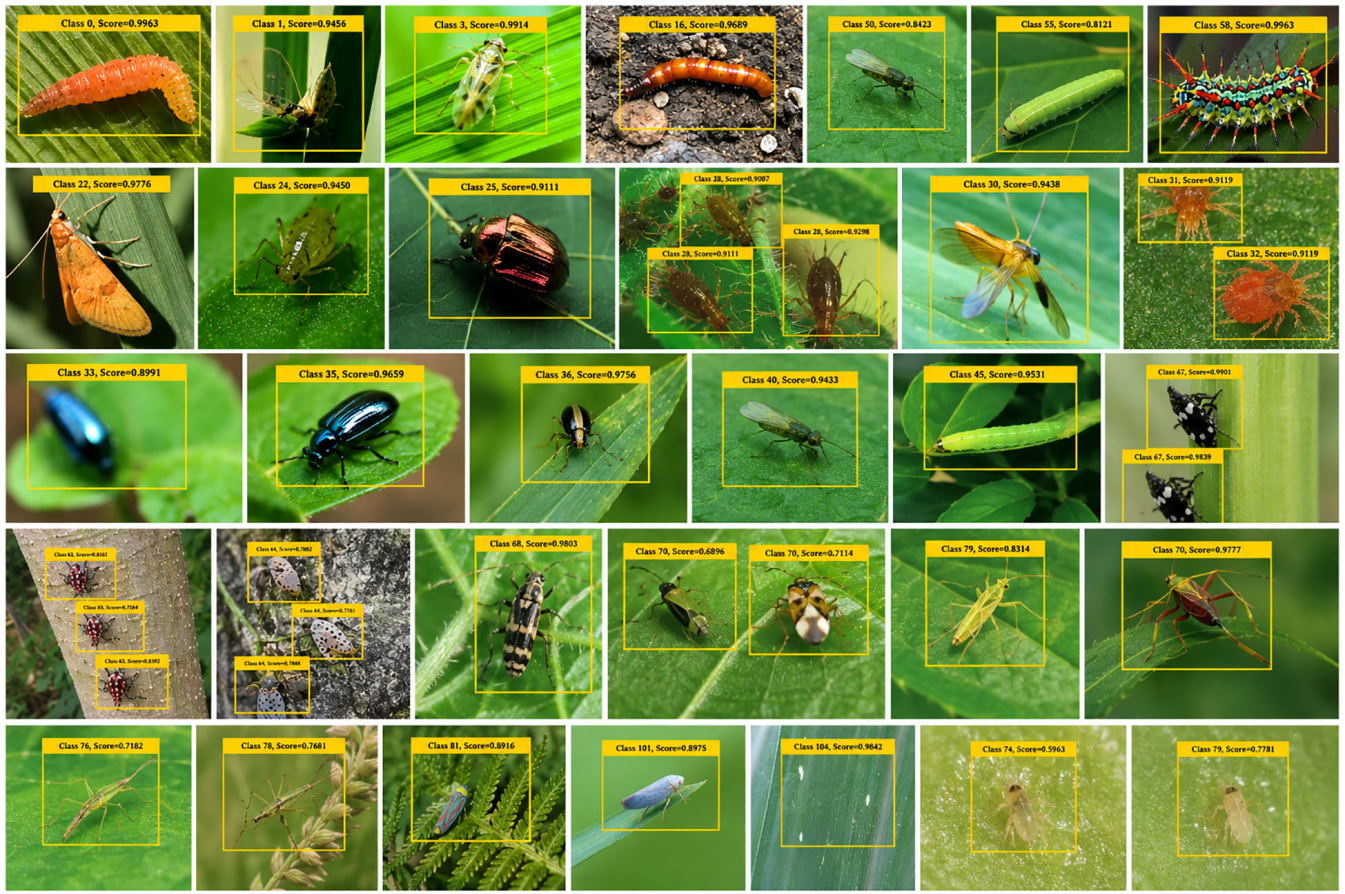
Localization results of PestRefineDet.

Accurate pest identification is essential for building an effective automated pest recognition system. To measure the localization performance of our framework, we conducted experiments using all test samples from the IP102 dataset, with visual results illustrated in **Figure 3**. The results indicate that our method can consistently localize pests of diverse sizes, shapes, and colors. Moreover, the proposed approach demonstrates effective performance on the challenging IP102 dataset, which includes complex backgrounds and variations in lighting, object orientation, and image acquisition conditions. The localization capability of our approach, facilitated by feature approximation, enables effective and precise identification and differentiation of pests across different categories. We quantitatively computed the localization performance by applying the mean Average Precision (mAP) and Intersection over Union (IOU). These metrics gauge the model’s proficiency in both localization and recognition across multiple pest groups. To perform localization, we used an IOU threshold of 0.5, designating regions with an overlap score less than this value as background and those exceeding it as pests. The model has attained a mAP and mean IOU values of 0.8361 and 0.8442, respectively. These results indicate that our method can reliably detect and accurately localize pests, even amidst diverse backgrounds.

**Figure 4:**
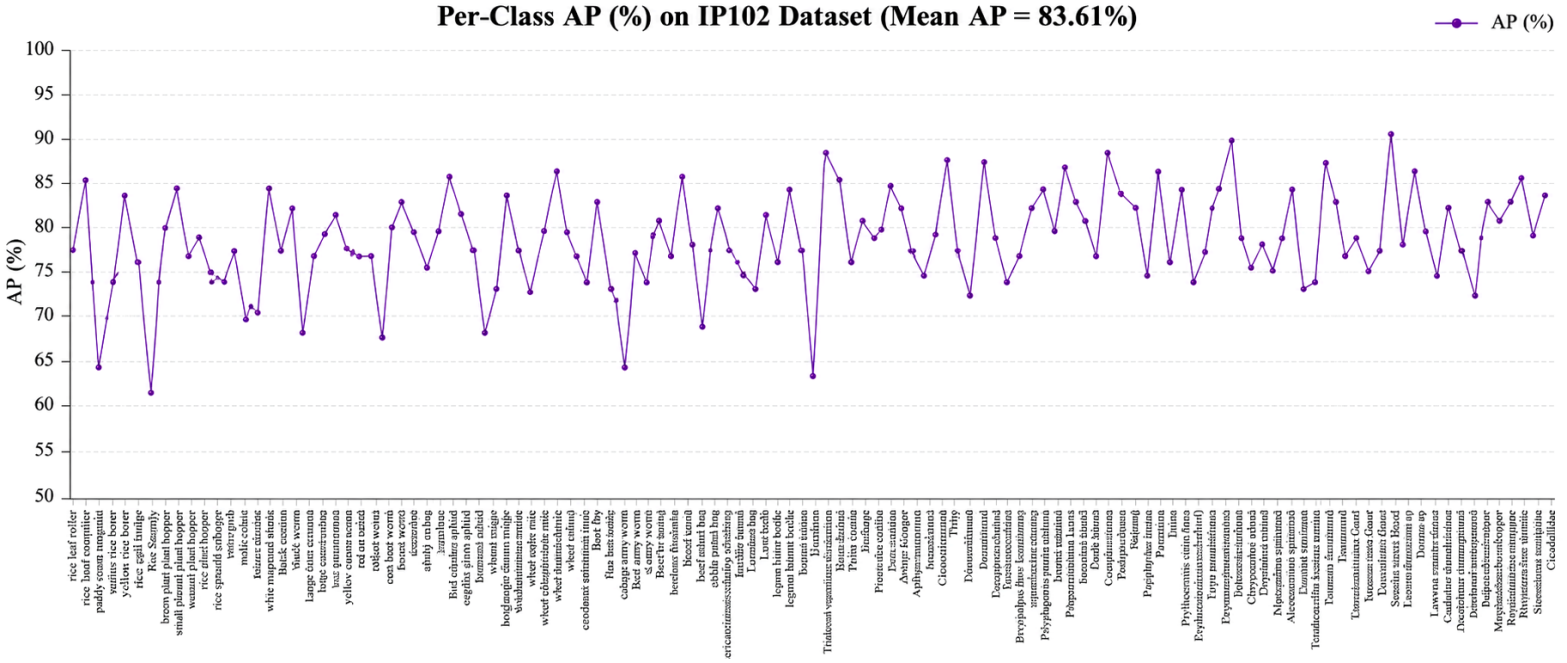
Per-class attained AP-score by the proposed approach.

To further analyze the effect of class imbalance in the IP102 dataset, **Figure 4** presents the per-class AP achieved by the proposed PestRefineDet across all 102 pest categories. The results indicate noticeable variations in detection performance among different classes, which is due to the inherent imbalance of the dataset. Pest categories with a larger number of training samples generally achieved higher AP values, which shows the ability of the approach to learn more discriminative feature representations from sufficient examples. In contrast, several minority classes exhibited relatively lower AP values because of limited training instances, higher intra-class variability, and visual similarities with surrounding backgrounds or other pest species. No additional data augmentation or class-balancing strategy was employed, as the objective was to evaluate the inherent feature learning capability of the proposed architecture under the official IP102 benchmark protocol. Despite this challenging setting, the dense feature reuse and enhanced gradient propagation of the DenseNet-41 backbone enabled the framework to learn robust multi-scale representations, resulting in stable performance across most pest categories. Most classes achieved AP values above 75%, while the overall mean AP reached 83.61%, demonstrating that the proposed PestRefineDet effectively generalizes across diverse pest classes despite the severe class imbalance of the IP102 dataset.

### 4.4 Crop-wise analysis of 102-class pest recognition performance

After evaluating the primary 102-class pest detection and recognition performance on the IP102 benchmark, an additional crop-wise analysis was conducted to investigate the model’s behavior across different agricultural categories. It should be noted that this analysis does not represent an independent classification task; rather, it groups the original pest categories according to their associated crop types to provide a more application-oriented interpretation of the results. The proposed PestRefineDet model was trained using the original 102 pest categories of IP102, and all quantitative evaluations were performed at the pest-category level. The crop-wise analysis was conducted only by aggregating the predictions of pest classes belonging to eight major crop groups, including rice, corn, beet, wheat, alfalfa, mango, citrus, and vitis.

**Figure 5:**
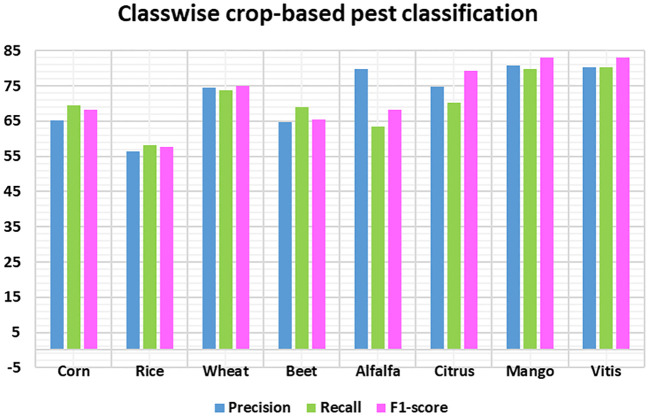
Class-wise crop-based pest classification performance of the proposed method.

**Figure 5** illustrates the crop-specific pest classification results of our method, evaluated by applying recall, precision, and F1 score. Across all crop-specific insect classes, the proposed framework achieved a precision of 83.9%, a recall of 82.3%, and an F1 score of 83.57%. This better performance is due to the effectiveness of our feature computation strategy, which provides discriminative and reliable representations for each pest class. Consequently, our customized RefineDet model demonstrates strong capabilities in crop-wise pest recognition, highlighting the efficacy of the approach.

We further evaluated the model’s performance by analyzing classification accuracies across the eight crop-specific pest categories, highlighting the variation in accuracy among different classes. The model achieved average accuracies of 78.68%, 89.19%, 79.57%, 83.49%, 69.59%, 91.28%, 87.17%, and 86.96% for rice, corn, beet, wheat, alfalfa, mango, citrus, and vitis, respectively. Overall, the mean classification accuracy was 83.57%, with consistently low error rates across all categories, demonstrating the strength of our approach. The method showed particularly strong performance for mango, vitis, and citrus, while rice and alfalfa exhibited lower accuracies because of visual resemblances with the background and high intra-class variability.

### 4.5 Ablation study

To investigate the effectiveness of the proposed DenseNet-41 integration within the RefineDet framework, an extensive ablation study was conducted. The objective of this analysis was to evaluate the contribution of different backbone networks while keeping the remaining detection framework, training strategy, and experimental settings unchanged. Several widely adopted backbone architectures, including VGG-16, ResNet-50, ResNet-101, EfficientNet-B0, and DenseNet-121, were integrated into the RefineDet framework and compared with the proposed PestRefineDet model. The comparison was performed based on the number of trainable parameters, model size, mAP, and recognition accuracy. The obtained results are presented in **Table 3**. Since PestRefineDet is an object detection framework, mAP@0.5 was considered the primary evaluation metric, where a detection was considered correct when the predicted bounding box achieved an IoU threshold of 0.5 with the ground-truth annotation. In addition, classification accuracy was reported as a supplementary metric to analyze the pest category recognition capability of different backbone networks. The comparison was performed in terms of mAP@0.5, classification accuracy, number of trainable parameters, and model size. The obtained results are presented in **Table 3**.

**Table 3:** Ablation study conducted for PestRefineDet.

Model	Backbone	Parameters (M)	Model size (MB)	mAP@0.5 (%)	Accuracy (%)
RefineDet	VGG-16	35.6	142.4	50.63	52.38
RefineDet	ResNet-50	29.5	147.2	65.44	68.82
RefineDet	ResNet-101	55.4	221.6	64.13	66.17
RefineDet	EfficientNet-B0	16.3	65.2	71.44	73.84
RefineDet	DenseNet-121	13.2	52.8	74.91	78.63
**PestRefineDet (Proposed)**	DenseNet-41	11.4	45.6	83.61	83.57

Bold values represent the performance of the proposed PestRefineDet framework.

The results demonstrate that the selection of the backbone network has a significant impact on pest detection performance. The conventional VGG-16-based RefineDet achieved a mAP@0.5 of 50.63%, indicating limited capability in extracting discriminative features from complex pest images containing small objects, background variations, and fine-grained visual similarities. The ResNet-50 backbone improved the detection performance by achieving a mAP@0.5 of 65.44% due to enhanced feature propagation through residual connections. However, further increasing the depth to ResNet-101 resulted in a slightly lower mAP@0.5 of 64.13%, indicating that deeper architectures do not necessarily provide improved generalization for challenging agricultural pest detection tasks.

The EfficientNet-B0 backbone achieved a mAP@0.5 of 71.44% while maintaining a lower computational complexity, demonstrating the advantage of efficient feature scaling. Similarly, DenseNet-121 improved the detection performance to 74.91% by exploiting dense feature connectivity, which facilitates feature reuse and improves information flow across network layers.

The proposed PestRefineDet framework with DenseNet-41 achieved the highest detection performance, obtaining a mAP@0.5 of 83.61% and a classification accuracy of 83.57%, while requiring only 11.4 million parameters and 45.6 MB storage space. This demonstrates that DenseNet-41 provides an effective balance between discriminative feature learning and computational efficiency. The improved performance can be attributed to the dense connectivity mechanism, which enables effective reuse of low-level and high-level feature representations and preserves important visual characteristics required for distinguishing visually similar pest categories. Furthermore, integrating DenseNet-41 with the ARM and ODM modules of RefineDet enhances feature representation, resulting in improved localization and classification performance.

Overall, the ablation study confirms that the proposed DenseNet-41 integration improves the detection capability of the RefineDet framework while reducing model complexity. The results demonstrate that PestRefineDet provides an effective and lightweight solution for fine-grained agricultural pest detection on the challenging IP102 benchmark dataset.

### 4.6 Comparison with other detection models

We performed a relative analysis of our proposed approach with several state-of-the-art object recognition methods to evaluate pest localization accuracy. Precise localization is particularly important in scenarios with noisy backgrounds or multiple pests, as misidentifying background elements can negatively impact classification performance. Accurate localization improves classification by excluding irrelevant background information. To assess this, we compared our approach with Faster R-CNN, SSD, YOLOv3, and RefineDet using different backbone networks as stated in (Albattah et al., 2023).

Our evaluation, performed on the IP102 dataset, assessed these models’ pest localization capabilities under various challenging conditions like complex backgrounds, noise, varying light, and changes in color, size, and shape. We used the mAP as a standard metric for performance analysis and also evaluated the computational complexity of each model. Results in **Table 4** demonstrate the superior results of our model for pest detection in comparison to others. Notably, models with powerful backbones like DenseNet generally exhibited better performance in pest recognition. Two-stage detectors, like Faster R-CNN, showed lower performance and higher computational complexity due to their reliance on anchor boxes for region of interest identification. In contrast, one-stage networks such as SSD, YOLOv3, and RefineDet (proposed) showed better performance by directly determining object position and category. However, their original implementations struggled with intense light variations, affecting pest recognition and localization accuracy. Our PestRefineDet model with DenseNet-41 as the backbone efficiently addressed the limitations of other methods. The DenseNet backbone facilitated learning of more representative features, enhancing pest localization and classification.

**Table 4:** Comparison with other object detectors.

Method	Backbone	mAP (%)	Test time(s/img)
Faster RCNN	VGG16	47.87	0.45
	DenseNet-100	48.12	0.41
SSD	VGG16	50.64	0.36
	DenseNet-100	50.95	0.33
YOLO	Darknet-53	50.64	0.29
	DenseNet-100	50.95	0.27
RefineDet	VGG16	65.12	0.31
**Proposed (RefineDet)**	**DenseNet-41**	**83.61**	**0.22**

Bold values represent the performance of the proposed PestRefineDet framework.

### 4.7 Comparison with state-of-the-art approaches

In this section, we compare the classification performance of our proposed approach with previous studies (Ayan et al., 2020; Nanni et al., 2020; Albattah et al., 2023; Ali et al., 2023; Anwar and Masood, 2023; Rimal et al., 2023; Wang et al., 2025; Agrawal et al., 2026) on the IP102 dataset. **Table 5** summarizes the pest classification results in terms of average accuracy. Ayan et al. (2020) employed CNN architectures (Inception-V3, Xception, and MobileNet) combined through an ensemble method, GAEnsemble, to improve classification performance. Similarly, the approach in (Nanni et al., 2020)integrated CNNs with a saliency-based technique to form an ensemble of classifiers, using a fusion-sum strategy at the output layer. While these methods achieved accuracies of 67.13% and 61.93%, respectively, their computational efficiency was limited due to the additional overhead of ensemble weight calculations. Further, a method for object detection was studied by Albattah et al. (2023) in order to identify and locate different kinds of pests from digital photos. For this, an enhanced CornerNet technique was presented in this study, whereby the DenseNet-100 has taken the role of the traditional feature extractor in order to extract the dense information from the samples, and attained an accuracy of 68.74%. In order to identify the crop pest from the photos, Ali et al. (2023) presented an enhanced Faster-RCNN model, which they named the Faster-PestNet, a DL technique. In this work, the Faster-RCNN model’s MobileNet feature extractor was used to pull out the dense information and reported an accuracy of 82.43%. The method enhances the outcomes of pest recognition, but it is unable to handle distorted samples. The idea of an ensemble network for crop pest categorization was put up by Anwara et al (Anwar and Masood, 2023). Three models, i.e., a 16-layered 19-layered VGG model and the ResNet50 approach, were combined into one. The classification assignment was completed by combining the features from all three models. The method is effective in identifying various pest species, but it has a high computational cost. Another framework of this kind was proposed in (Rimal et al., 2023), where a DL method known as the MobileNet framework was applied to the classification of various crop pest types. This method demonstrates an effective way to identify agricultural pests, but more work needs to be done to increase recognition accuracy. Further, Agrawal et al. (2026) suggested a DL approach for recognizing pests from the input images by utilizing structured DensNet approach and attained an accuracy value of 82.69%, however, lacks to locate the exact location of the pests on the samples. Next, in (Wang et al., 2025), an improved EfficientNetb0 approach was presented for pest classification, and attained an accuracy score of 74.06%, which needs further enhancement.

**Table 5:** Comparison with existing techniques.

Reference	Accuracy %
Ayan et al. (2020)	67.13
Nanni et al. (2020)	61.93
Albatah et al (Albattah et al., 2023)	68.74
Ali et al. (2023)	82.43
Anwar et al (Anwar and Masood, 2023)	82.50
Rimal et al. (2023)	82.04
Agrawal et al. (2026)	82.69
Wang et al. (2025)	74.06
**Proposed**	**83.57**

Bold values represent the performance of the proposed PestRefineDet framework.

Our proposed model with a DenseNet-41 base network overtakes these works by attaining an average accuracy of 83.57%. Notably, DenseNet is capable of effectively computing feature maps by joining output from previous layers as input to all subsequent layers. These calculated features are utilized by the RefineDet model, significantly enhancing model performance for the task of pest recognition on the challenging IP102 dataset. Furthermore, our technique demonstrates computational efficiency and robustness, enabling more precise insect identification compared to existing methods. Consequently, the proposed framework demonstrates promising potential for supporting automated pest monitoring applications; however, further validation using field-acquired images is required before practical deployment.

### 4.8 Practical implications and deployment feasibility

Although the proposed PestRefineDet framework is evaluated using the benchmark IP102 dataset, its design characteristics indicate potential suitability for practical agricultural pest monitoring applications. In real-world farming environments, pest identification is a critical step in integrated pest management (IPM), where timely and accurate detection can support targeted control strategies and reduce unnecessary pesticide applications. Automated image-based pest recognition systems can assist farmers and agricultural experts by providing rapid pest identification and enabling early intervention decisions.

The computational efficiency of the proposed framework is an important factor for practical deployment. The proposed DenseNet-41 backbone achieves improved recognition performance while maintaining a reduced number of parameters and model size compared with deeper backbone architectures. This lightweight design reduces memory requirements and computational overhead, making PestRefineDet more suitable for deployment on resource-constrained platforms such as edge devices, mobile agricultural monitoring systems, and smart farming platforms. Recent studies have demonstrated the feasibility of deploying lightweight deep learning-based pest detection models on edge devices for real-time agricultural monitoring, highlighting the importance of balancing detection accuracy with computational efficiency.

Furthermore, the impact of detection errors should be considered when applying AI-based pest monitoring systems in agricultural decision-making. False negatives (missed pest instances) can delay intervention and allow pest populations to increase, whereas false positives can result in unnecessary inspections or localized treatment actions. Therefore, practical deployment should consider confidence thresholds, expert verification, and integration with other agricultural information sources, like crop growth stage, environmental conditions, and pest population dynamics, before recommending control measures.

Within an integrated pest management framework, PestRefineDet can serve as an intelligent monitoring component by providing rapid pest recognition and localization information. The generated detection results can assist agricultural specialists in selecting appropriate and targeted management strategies rather than relying on excessive preventive pesticide applications. However, further validation using field-collected images under diverse environmental conditions, including variations in illumination, background complexity, and crop growth stages, is required before large-scale deployment.

## 5 Conclusion

In this study, we proposed a cost-effective deep learning technique for automated detection and classification of crop pests in field environments using drone imagery. The approach is built around a customized RefineDet model, utilizing DenseNet-41 as the backbone network for extracting discriminative features from input images. The refined anchors and features enable accurate identification of diverse pest types. Evaluation was performed on the IP102 dataset, a challenging benchmark containing in-field pest images, demonstrating the practical effectiveness of our method. Extensive experiments showed that the framework can accurately localize and classify pests across multiple categories, even under complex backgrounds and variations in pest characteristics such as shape, color, size, orientation, and illumination. The proposed PestRefineDet framework provides a promising foundation for intelligent pest monitoring systems by combining accurate pest recognition with computational efficiency. Although the current evaluation is based on the IP102 benchmark, future work will focus on field-scale validation and integration with real-time agricultural decision-support platforms for practical IPM applications.

## Data Availability

The original contributions presented in the study are included in the article/supplementary material. Further inquiries can be directed to the corresponding authors.
